# Supplementary material for: In-Silico Analysis of pH-Dependent Liquid-Liquid Phase Separation in Intrinsically Disordered Proteins
Source: Biomolecules. 2022 Jul 12;12(7):974. doi: 10.3390/biom12070974 (PMC9313257; doi:10.3390/biom12070974)
Supplement: Supplementary file 1 [file biomolecules-12-00974-s001.zip › biomolecules-1806347 supplementary update.pdf]

Supplementary material for

In-silico analysis of pH-dependent Liquid-Liquid Phase Separation in Intrinsically Disordered Proteins

Carlos Pintado-Grima [1†], Oriol Bárcenas [1†] and Salvador Ventura [1,*]

Mann-Whitney-Wilcoxon two-sided test

Mann-Whitney-Wilcoxon test is a non-parametric statistic test that analyzes differences between two different samples, contrasting whether they proceed from different populations. It does this through ordering all the datapoints from smaller to greater and analyzing if the observations are randomly intercalated (thus pointing towards equal distributions among populations), or not.

We used this approach instead of a t-test, which is also common to compare mean differences between populations, because the latter is more restrictive and needs a series of assumptions to be applied on data: independence, normality and homoscedasticity (same variance).

Wald Test

Wald test is a parametric statistic test that has numerous applications. When applied to linear regression, it can be used to reject the null hypothesis. In this case, it would be that the slope is 0 (the dispersion does not follow a linear regression tendency). Statistical significance, like the one obtained in this case, would allow for the rejection of the null hypothesis; this meaning that linear regression can be used to fit the analyzed data.
